# Medical Students’ Reflections on Racism in German Healthcare and Its Alignment With National Data: A Qualitative Case Study Using the Example of Muslim Patients

**DOI:** 10.7759/cureus.83662

**Published:** 2025-05-07

**Authors:** Arian Mauntel, Utz Settmacher, Aysun Tekbaş

**Affiliations:** 1 Department of General, Visceral and Vascular Surgery, Jena University Hospital, Friedrich Schiller University Jena, Jena, DEU; 2 Research Programme “Advanced Clinician Scientist Programme”, Interdisciplinary Center of Clinical Research, Jena University Hospital, Friedrich Schiller University Jena, Jena, DEU

**Keywords:** cultural and religious sensitivity, intercultural competence, nadira report, personalized medicine, racism and discrimination in german health care system

## Abstract

Background

Racism in the German healthcare system has received increasing attention, yet its integration into medical education remains limited. Systemic disparities continue to affect racially marked individuals and cultural minorities, underscoring the need to explore how these issues are perceived by future healthcare professionals. This study examines how medical students understand racism and intercultural competence in clinical settings, using the example of Muslim patient care, and compares their perspectives with national-level developments.

Methods

A qualitative case study was conducted with 65 medical students enrolled in an elective course on ethical aspects of caring for Muslim patients. Participants responded to open-ended questions regarding their motivations, clinical experiences, and educational goals. Responses were analyzed using structured qualitative content analysis (MAXQDA 24©, VERBI, 2024).

Results

Ten central themes emerged, including communication barriers, limited awareness of culture- and faith-specific practices, and the importance of individualized care. While racism and discrimination were acknowledged, they were addressed less frequently. The reflections aligned with broader observations on structural inequalities and the need for culturally sensitive care.

Conclusion

The findings reveal notable gaps in intercultural competence among future physicians, with implications for both clinical care and medical education. Students identified relevant challenges but lacked tools to address them confidently. Although preliminary, these results highlight the need to further integrate topics such as racism, religious diversity, and structural discrimination into medical training. Doing so may better prepare healthcare professionals to deliver equitable and patient-centered care in an increasingly diverse society.

## Introduction

The World Medical Association (WMA), which represents more than 10 million physicians worldwide, formally recognized the existence of racism in medicine during its 2022 General Assembly in Berlin, Germany [1]. The assembly adopted the Declaration of Berlin on Racism in Medicine to condemn all forms of racism and clarify that physicians must provide care without any bias or engagement in discriminatory conduct due to race or any other factor. This marks a significant initial step towards Germany acknowledging and addressing the issues of racism within its healthcare system. However, the German debate on racism in medicine is rather underdeveloped [2]. It has been historically overlooked in German medical education, and discussions have largely been confined to the context of medicine during the National Socialist era [3]. Until recently, comprehensive data and detailed analyses on the issue of racism in the German healthcare system were lacking [4]. However, in light of recent antiracist movements, professional networks and student initiatives are bringing attention to the disparity between the national conversation and the broader international discourse [5].

Studies highlight the disadvantages faced by specific patient groups within the healthcare system [6]. Possible reasons are provided by Schoedwell et al., who conducted a study with 112 hospital employees in Berlin, examining how economic conditions contribute to structural discrimination and racism in hospital care [7]. Limited resources and insufficient funding for language services overburden healthcare workers, leading to culturalization, where patients' behaviors are attributed to their "culture," and open racism. This is supported by Ramsak et al., who examined the experiences and attitudes of healthcare professionals regarding social diversity and equal access to healthcare in Croatia, Germany, Poland, and Slovenia [8]. Key challenges identified include healthcare underfunding, language barriers, insufficient cultural training, and lack of institutional support. While most interviewees did not see systemic exclusion of minority groups, they reported instances of individual discrimination, such as homophobia and racism.

Data on individuals affected by discrimination within the German healthcare system are provided by a cross-sectional online survey of 2,201 adults conducted by von dem Knesebeck et al. [9]. A total of 26.6% reported experiencing discrimination in healthcare, with the most frequent reasons being health issues or disability (15%), age (9%), socio-economic status (8.9%), and racism (4.1%). Younger individuals, women, second-generation migrants, and those with low income were more likely to report discrimination within the healthcare system, with women, younger age groups, and those experiencing racism being at particular risk [9].

A pivotal insight into the issue of racism within the German healthcare system is provided by the recent NaDiRa Report 2023 (National Discrimination and Racism Monitor), “Racism and its symptoms” [10]. For the monitoring report, researchers from the DeZIM Institute (Deutsches Zentrum für Integrations- und Migrationsforschung) conducted a comprehensive representative survey from June to November 2022 with 21,000 participants to examine their discrimination experiences through a combination of quantitative and qualitative research methods. The main findings are that discrimination and racism are widespread, varying by social group, characteristics, and context. These issues are commonly reported in healthcare, impacting not only those directly targeted but also exerting broader societal effects. Among the groups studied, Black individuals experience the highest levels of overt discrimination, while racially marked individuals predominantly face bias based on race rather than ability, gender, or class. A total of 35% of Muslim women report unfair treatment by medical staff, with the figures rising to 39% for Black women and 29% for Asian women. Additionally, 13-14% of Black, Asian, and Muslim women, along with 8% of men in these groups, have avoided or delayed medical treatment due to fear of discrimination. People with names common in Nigeria or Turkey are less likely to receive positive responses to appointment requests compared to those with German-sounding names. The report also highlights that frequent experiences of discrimination are associated with increased anxiety, depressive symptoms, and diminished trust in the healthcare system among these individuals [10].

In terms of medical education, the participatory exploratory analysis revealed that there is an underrepresentation of racially marked patient groups in teaching materials, and these groups are often depicted as deviations from the norm. The report criticizes that the professional self-image of physicians hinders reflection on racism [10].

To address these issues, we need evidence-based, context-sensitive approaches within German medical education. Online focus group discussions reveal that there are varied levels of knowledge, awareness, perceptions, and specific learning needs among medical students regarding structural racism and intersectionality [11,12].

The topic continues to gain further scientific and political significance due to the ongoing development of the National Competence-Based Learning Objectives Catalogue for Medicine (NKLM), which emphasizes the importance of recognizing and addressing ethical, social, cultural, legal, and historical aspects in medical care contexts [13]. A key learning objective is to identify and combat discrimination and stigmatization based on race, ethnicity, gender, religion, disability, age, or sexual identity, aiming to prevent and eliminate such disadvantages [13].

As academic staff, we are particularly committed to developing new teaching concepts focused on intercultural competencies to address the current social and political needs in medical education. As a first step, we introduced a curriculum specifically tailored to understanding the ethical and religious particularities of Muslim patients in a hospital setting. This is because up to 30% of medical cases in certain healthcare settings involve individuals of the Islamic faith [14]. The learning objectives include conducting medical procedures with consideration of ethico-religious factors and demonstrating tailored patient care. Furthermore, in light of the findings from the NaDiRa Report, we were interested in understanding where students themselves perceive current medical-ethical issues, as this could potentially motivate their participation in such courses. Thus, a survey of the course participants was conducted before the start of the course. It is important to emphasize that religion is an integral part of culture; religious values, practices, and identities often shape cultural norms and patient expectations [15]. Muslim individuals are frequently subject to processes of racialization in the healthcare system, meaning they are perceived and treated through the lens of cultural or racial stereotypes based on name, dress, or phenotypic features [10]. This results in discrimination structurally similar to racism. As such, student reflections often refer not only to religious aspects but also to racialized treatment and bias. This qualitative analysis, therefore, aims to provide insights into the students' current knowledge regarding discrimination and intercultural competencies within the German healthcare system, as well as identify the gaps that need to be further addressed through medical education. We seek to determine how the perspectives of patients, as highlighted by the NaDiRa Report [10], correlate with the viewpoints of the students.

## Materials and methods

This study employs a qualitative case study approach to provide an in-depth understanding of medical students' perspectives on culturally and religiously sensitive care, including reflections on perceived racism and discrimination. Participants were eligible if they were enrolled in the elective course “Medical ethical aspects in dealing with Muslim patients” at Jena University Hospital and had completed at least the sixth semester of their medical studies. Students were excluded if they failed to complete the questionnaire or withdrew consent. Between January and June 2023, 65 students voluntarily provided informed consent and participated in the study.

Before the course commenced, participants were invited to respond to four open-ended questions hosted on the Moodle® platform. The questions covered students’ motivations for course participation, prior experiences with Muslim patients, perceived ethical challenges in clinical practice, and personal learning objectives.

**Figure 1:**
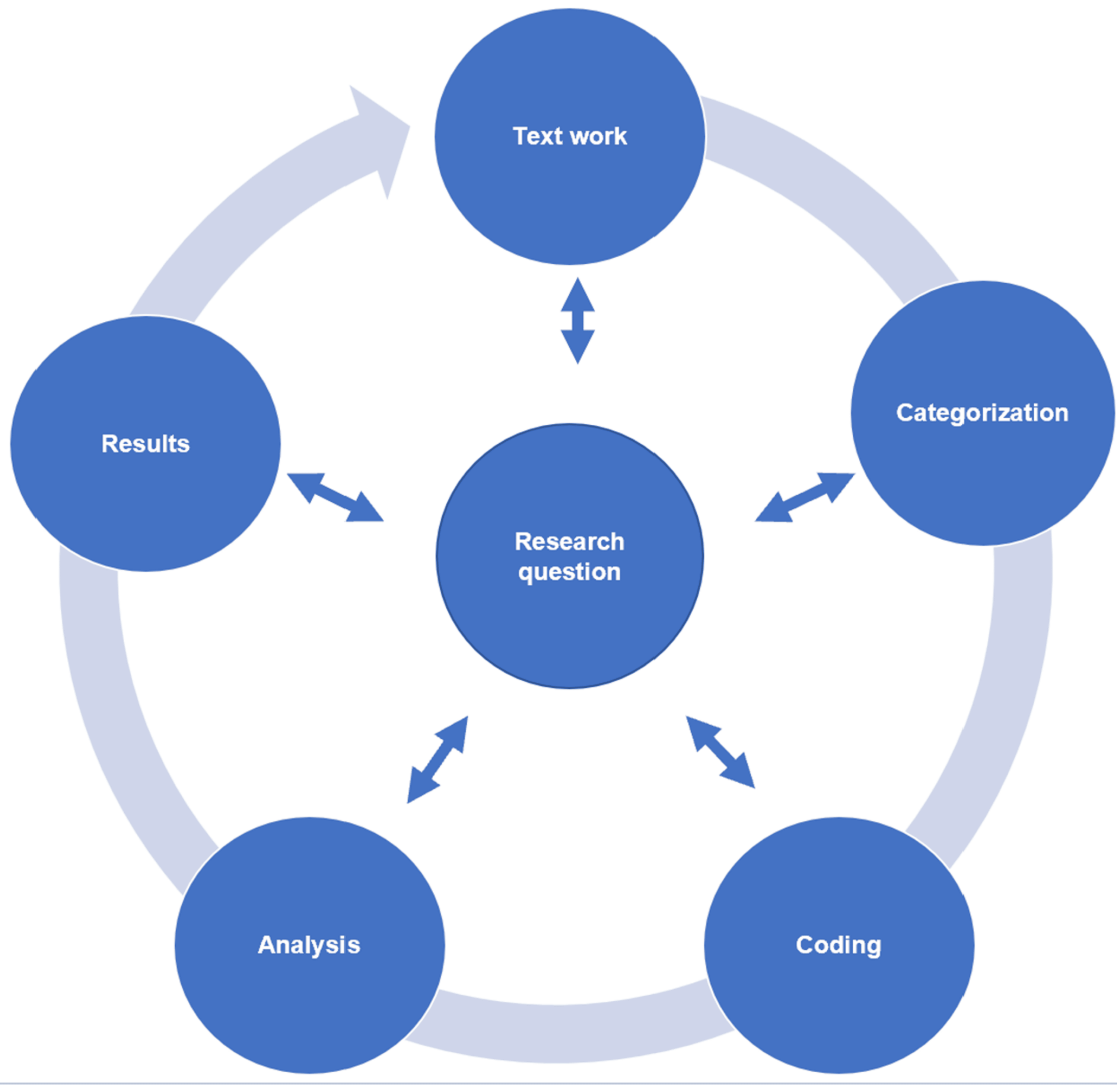
Procedure for content analysis of students' responses, based on Mayring. [16]

Responses were submitted as free-text entries and analyzed using a structured qualitative content analysis following Mayring’s approach [16], supported by the software MAXQDA 24© (VERBI, 2024) (Microsoft, Redmond, USA). This method follows a systematic, rule-based procedure that aligns closely with the research question and allows for iterative refinement of categories throughout the analysis process. The process involves iterative text work, inductive categorization (themes emerge from the data itself), coding, analysis, and interpretation, ensuring methodological transparency and comprehensibility (Figure 1). Coded segments were analyzed to identify key patterns and insights. To ensure reliability, a second researcher independently coded a representative sample of responses. Interrater reliability was calculated using Cohen’s Kappa, yielding a value of 0.75, indicating substantial agreement.

In addition to qualitative data, demographic information, including age, gender, semester, and religious affiliation, was collected and presented as percentages.

## Results

Demographic data

The demographic data is presented in Table 1. Most of the students were female (73.9 %), in their sixth semester (61.5 %), and between 20 and 25 years old (90.8 %). The majority did not belong to Islam (93.8 %). A total of 66% of the students reported having prior experience with Muslim patients in the context of their practical training during their studies.

**Table 1:** Demographic data of the 65 participating students. M: Male, f: Female

Parameter	%
Gender	
m	24.6
f	73.9
non-binary	1.5
Semester	
6^th^	61.5
7^th^	15.4
8^th^	3.1
9^th^	15.4
≥10^th^	4.6
Age	
20 - 25	90.8
26 - 30	6.2
≥30	3
Religious affiliation	
Islam	6.2
Other	93.8

Coding

**Figure 2:**
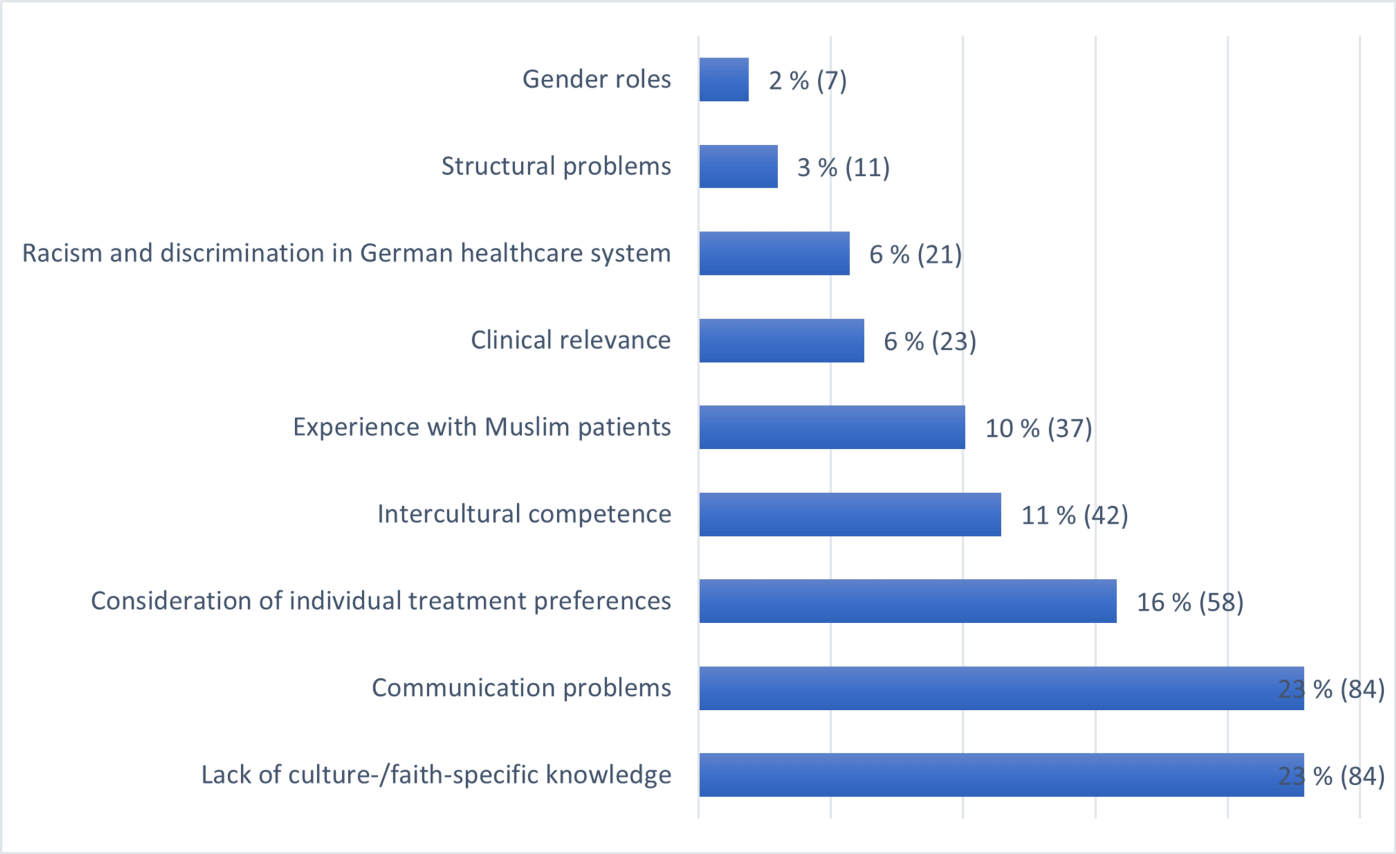
Proportion of distinct codes relative to the total number of coded segments.

All four questions were thoroughly answered by the 65 study participants. Following Mayring's method, 367 coded segments were generated from the responses. These segments were subsequently categorized into nine distinct codes. The reliability check revealed a Cohen’s Kappa of 0.75. The codes are presented in Figure 2 and include the following: 1. Communication problems. 2. Lack of culture-/faith-specific knowledge. 3. Consideration of individual treatment preferences. 4. Intercultural competence. 5. Experience with Muslim patients. 6. Clinical relevance. 7. Racism and discrimination in the German healthcare system. 8. Structural problems. 9. Gender roles.

Content analysis according to the assigned codes

Communication Problems

The key themes that emerge from the text passages are the need to improve communication and understanding when treating Muslim patients: “I want to learn more about the communication gaps.” (St6.3) Specific areas of concern include language barriers, maintaining patient privacy and modesty, building trust and rapport, and navigating potential differences in medical beliefs and practices.

Lack of Culture-/Faith-Specific Knowledge

The students reported a lack of culture- and faith-specific knowledge, particularly when caring for Muslim patients. Many respondents expressed a desire to learn more about culture, customs, traditions, and beliefs in order to provide more sensitive, respectful, and appropriate medical care. The knowledge gaps include understanding gender roles, dietary restrictions, modesty requirements, and religious rituals that may impact medical treatment: “I want to learn more about cultural backgrounds that are important for the future treatment of patients and also about the specific aspects I need to pay attention to so that the patients feel comfortable.” (St6.1) Respondents recognized that this knowledge gap can lead to misunderstandings, discomfort, and even rejection of care by Muslim patients.

Consideration of Individual Treatment Preferences

In relation to this topic, the key points from the text passages highlight the importance of recognizing and respecting the cultural backgrounds of patients to provide high-quality, personalized medical care: “It is important to me that my future patients, regardless of their background or religious beliefs, feel well cared for with me. To ensure this, I want to look beyond cultural differences and learn what I should be attentive to.” (St5.25)

Challenges often arise when medical recommendations need to be reconciled with patients' religious convictions. However, a flexible and empathetic approach, which prioritizes patient autonomy and promotes shared decision-making, is ideal. The overall goal is to deliver care that is sensitive to individual needs, ensuring that patients from all backgrounds feel comfortable, respected, and well-cared for.

Intercultural Competence

The participants highlight the importance of developing intercultural competence in healthcare to deliver culturally sensitive and effective care for patients from diverse backgrounds: “Culturally sensitive handling of patients in an increasingly globalized world is crucial.” (St6.6) A key theme is the necessity of understanding cultural beliefs, practices, and perspectives related to health, illness, and medical treatment. This understanding is crucial for overcoming communication challenges and building trust with patients from various cultural and religious contexts. The students also address the desire to learn strategies for navigating cultural differences and avoiding unintended negative impacts on patient care and outcomes.

Experience With Muslim Patients

The students reported that they encountered various experiences with Muslim patients during their clinical internships. Some faced challenges with patient compliance and cooperation, particularly when dealing with male Muslim patients who were unwilling to accept female healthcare providers. There were also instances where Muslim patients or their families were not receptive to certain medical practices or treatments due to specific belief systems: “I have reached my limits on more than one occasion because the patients and/or their relatives were not compliant, which was very frustrating for me.” (St5.21)

Clinical Relevance

The students indicated that the topic has high clinical relevance. Healthcare professionals often feel hesitant or unsure about how to communicate and provide care that aligns with the beliefs and preferences of patients. This can lead to a loss of information and a reduced quality of care: “We are making avoidable mistakes during treatment that negatively impact the outcome or the patient's trust. These often arise from a lack of knowledge.” (St5.14)

Respondents hope that increased knowledge and sensitivity will allow them to interact in a more thoughtful and appropriate manner, leading to better health outcomes: “There is a lack of training and education to raise awareness among staff about various forms of discrimination and to reflect on their own biases.” (St4.1)

Racism and Discrimination in the German Healthcare System

The students address the critical issues of racism and discrimination in healthcare: “Mutual acceptance of each other's cultural values is very important. Rigid opinions and the accompanying highly racist and discriminatory attitudes are very dangerous and impair medical treatment.” (St5.3). Additionally, the impact of making generalizations about patients rather than treating them as individuals is discussed, and how structural barriers and personal biases can further complicate patient interactions. Students observe the tendency to impose their own cultural values without understanding others. Misunderstandings about religious symbols and poor treatment, especially towards patients from Arab backgrounds, are also considered significant problems. Distrust between patients and providers, misinformed staff relying on stigmas, and a lack of awareness about Muslim patients' experiences in German hospitals contribute to these issues. As a result, patients may experience trauma, misdiagnoses, and may avoid seeking healthcare services.

Structural Problems

The students view structural problems in the healthcare system, such as time constraints, as a root cause of everyday medical ethical conflicts: “Often, there is not enough time to address potential individual needs in patient care (much of it is standardized).” (St4.16) These issues can lead to inadequate attention and care for patients, particularly those from diverse backgrounds. As a result, there may be conflicts and an inability to address individual needs or understand the specific challenges faced by Muslim or foreign patients in the healthcare system.

Gender Roles

The participants consider traditional gender roles as a challenge in healthcare, particularly regarding intimate examinations and treatments: “I can imagine that differing views on gender roles could become an issue when a female patient is treated by a male doctor or a male patient by a female doctor, particularly in situations involving intimate examinations.” (St5.11) This can be especially true for Muslim patients, where traditional gender roles and modesty norms may conflict with standard medical practices. Addressing these issues sensitively and accommodating patient preferences where possible is important to provide equitable, culturally competent healthcare.

In summary, the text analysis reveals that the main themes identified from the survey include communication problems, lack of culture-/faith-specific knowledge, and consideration of individual treatment preferences due to specific belief systems. These issues, which account for 59%, were highlighted based on students' motivations to participate in the seminar, their prior experiences, medical-ethical challenges, and personal learning objectives. Racism and discrimination in the German healthcare system were addressed in 6% of the responses (Figure 2). The students' statements further revealed their interest in various aspects of women's healthcare, particularly issues related to fertility and pregnancy termination. They also emphasized the need to consider complex topics such as embryo research, abortion, palliative care, and euthanasia.

## Discussion

In the context of increasingly diverse societies, a thorough understanding of racism and its impact on health is crucial for the future of medical practice. It is possible to draw parallels between our survey and the NaDiRa Report [10] in terms of content, with the NaDiRa Report primarily focusing on the patient perspective.

The students' perspectives highlight key issues in cultural and religious sensitivity within the German healthcare system, frequently mentioning racism and discrimination. They emphasize the importance of understanding and respecting patients' cultural and religious backgrounds to ensure high-quality care. Communication challenges, particularly with Muslim patients, and the need for enhanced cultural competence are stressed. Additionally, traditional gender roles and modesty norms are identified as difficulties in intimate medical situations, while structural issues such as time constraints may obstruct the addressing of individual patient needs and the development of intercultural competence. These findings strongly reflect the awareness of the need for culturally sensitive care among medical students, correlating with the findings of previous studies [7,8,10-12].

Our survey and the NaDiRa Report [10] reveal five key parallels in their findings (Table 2).

**Table 2:** 5 key parallels in our findings and the NaDiRa report. [10]

Common key subject	NaDiRa Report	Student’s comments
Prevalence of racism and discrimination	The widespread racism and discrimination in Germany, particularly against racially marked groups, are proven.	Racism and discrimination are major concerns within the German healthcare system.
Impact on specific groups	Data show that Black, Asian, and Muslim women are disproportionately affected by discrimination in healthcare, leading to delays in seeking treatment and negative health outcomes.	The students highlight the challenges faced by Muslim patients, emphasizing difficulties in communication and the need for greater religious sensitivity to enhance care.
Cultural sensitivity	Racially marked groups are underrepresented in medical education, and there is a lack of reflection on racism within the medical profession.	There is a desire to improve understanding of cultural and religious practices, recognizing the importance of cultural competence in delivering personalized and respectful care.
Structural issues	Discrimination leads to anxiety, depressive symptoms, and a lack of trust in the healthcare system among affected individuals.	Structural problems, such as time constraints within the healthcare system, hinder the ability to address individual patient needs and to develop intercultural competence.
Professional reflection	The lack of reflection on racism within the medical profession and the inadequate representation of racially marked patient groups in teaching materials are deeply concerning.	There is a need to learn more about cultural and religious differences in medical education to enhance medical practice.

Both reports underscore the widespread prevalence of racism and discrimination within the healthcare system, highlighting how these issues negatively impact patient care.

The reports show that certain groups, particularly racially marked individuals and cultural minorities, are disproportionately affected by discrimination, leading to unequal treatment and adverse health outcomes. The importance of cultural sensitivity in healthcare is emphasized in the NaDiRa Report and our survey, noting the need for healthcare providers to understand and respect diverse cultural backgrounds to deliver high-quality care. Structural problems within the healthcare system are identified in both studies, such as time constraints and systemic biases, that hinder the provision of personalized and culturally competent care. Institutional structures maintain racist conditions, often exacerbated by staff shortages, time constraints, high workloads, and rigid hierarchies [10]. Both documents highlight the need for greater reflection on racism within the medical profession and call for improvements in medical education, particularly regarding the representation of racially marked patient groups in teaching materials.

To address these challenges, the NaDiRa report provides targeted recommendations for policymakers and healthcare stakeholders. These practical steps aim to mitigate the issues effectively and create a more equitable and inclusive healthcare environment [10].

In the Anglo-American literature, there is a large number of recommendations for anti-racist interventions in medical education [17-19]. However, due to cultural differences, these recommendations are not directly transferable to the German context. Therefore, based on our findings, we recommend addressing the mentioned "common key subjects" and developing a corresponding, nationally applicable teaching concept.

Changes in the academic and clinical environments are essential, both at the individual and institutional levels. Institutional support measures should be systematically implemented to address racism during the academic training and to prevent it in clinical practice [11]. Training and resources are needed to help healthcare staff recognize and address their own biases and provide culturally competent care to patients from diverse backgrounds. Acquiring a better grasp of these factors is crucial for improving communication, building trust, and delivering more holistic, patient-centered care in diverse healthcare settings.

The study has a few limitations. It includes students who self-selected into an elective course on intercultural ethics, which may introduce response bias due to higher initial interest in the topic. The course specifically focused on Muslim patients; hence, reflections on other racially or culturally marked groups were less prominent. However, as outlined in the NaDiRa report [10] and other current literature, Muslim individuals in Germany often face racialization in clinical contexts, where they are perceived and treated not only based on religious affiliation but also racial or ethnic markers. Furthermore, the perspectives of other healthcare professions are also of interest but are not considered in this study.

Our study thus contributes to understanding how students perceive racialized discrimination in healthcare, highlighting the necessity of addressing these intersecting factors in medical education. It represents the largest sample of surveyed students on this topic in Germany to date and offers valuable insight for developing targeted educational modules.

## Conclusions

This study underscores the need to better integrate the topics of racism, cultural and religious diversity, and structural discrimination into the German medical curriculum. Medical students demonstrated high awareness of these issues and a strong desire for education that prepares them to meet the needs of a diverse patient population. While reflections directly naming racism were less frequent, the underlying concerns about unequal treatment and stereotyping closely align with national data.

We can conclude that the students' perspective correlates with the NaDiRa report and that key themes such as the prevalence of racism and discrimination in the German healthcare system, the impact on specific groups, cultural and religious sensitivity, structural issues, and the importance of professional reflection must be considered in curriculum development. By addressing both structural and interpersonal dimensions of bias, future educational initiatives can promote equity and trust in clinical practice. These insights offer a valuable contribution to the ongoing national debate on how best to prepare medical professionals for patient-centered care in an increasingly diverse society.
